# Zooplankters’ nightmare: The fast and efficient catching basket of larval phantom midges (Diptera: *Chaoborus*)

**DOI:** 10.1371/journal.pone.0214013

**Published:** 2019-03-22

**Authors:** Sebastian Kruppert, Lisa Deussen, Linda C. Weiss, Martin Horstmann, Jonas O. Wolff, Thomas Kleinteich, Stanislav N. Gorb, Ralph Tollrian

**Affiliations:** 1 Department of Animal Ecology, Evolution and Biodiversity, Ruhr-University Bochum, Bochum, Germany; 2 Department of Biological Sciences, Macquarie University, North Ryde NSW, Australia; 3 Department of Functional Morphology and Biomechanics, Kiel, Germany; Texas A&M University System, UNITED STATES

## Abstract

Filter feeding zooplankton are a crucial component of limnic food webs. Copepods and cladocerans are important prey organisms for first-level predators like the common and abundant larvae of phantom midges (*Chaoborus* sp.). The latter possess a complex catching basket built of head appendages specialized to capture small crustaceans. The predator-prey-relationship of *Chaoborus* (Diptera, Nematocera) and *Daphnia* (Crustacea, Cladocera) has been studied in particular detail owing to the daphniids’ ability to react upon the threat of predation with inducible defenses. *Daphnia pulex* expresses so-called ‘neckteeth’ in the presence of *Chaoborus* larvae that are discussed as a defensive trait that interferes with the larval head appendages and their effectiveness has been shown in several studies. Nonetheless, mode of function of these neckteeth is not understood and the hypothesis that they interfere with the predator’s head appendages still has to be confirmed. To clarify the role of neckteeth in *Daphnia*, an understanding of the *Chaoborus* capture apparatus is essential. Here, we present a detailed three-dimensional analysis of *Chaoborus obscuripes’* larval head morphology as well as a kinematic analysis of the attack motion, which revealed an impressive strike velocity (14 ms to prey contact). The movement of the larvae’s head appendages is reconstructed in the three-dimensional space using a combination of high-speed videography, micro-computed tomography and computer animation. Furthermore, we provide predation trial data to distinguish between pre- and post-attack defensive effects in *D*. *pulex*. Our findings suggest a combination of pre- and post-attack defenses with an average effectiveness of 50% each. With this study, we quantitatively describe prey capture kinematics of *C*. *obscuripes* and take a further step to reveal the neckteeth’ mode of function in *D*. *pulex*.

## Introduction

Most lentic ecosystems are organized in multi-level food webs. Herbivorous zooplankters, feeding on primary producers, are the first level of consumers followed by first-level predators. One prominent example of a first-level predator in limnic ecosystems is the larva of the phantom midge (Diptera, Nematocera, *Chaoborus* Edwards). *Chaoborus* larvae hatch from eggs deposited in freshwater habitats. They develop through four larval instars before they pupate and leave the water to mate and reproduce [1]. As *Chaoborus* is a typical inhabitant of freshwaters world-wide, these larvae can be regarded as a central component of aquatic food webs [2]. While they serve as an important food source for higher trophic levels including many fish species, the larvae themselves prey on ciliates, copepods, and cladocerans [3]. They are ambush predators that hold their position in the water column using two pairs of tracheal gas sacs for buoyancy correction [4]. *Chaoborus* larvae detect prey with the help of mechano-sensory sensillae [5–7]. Prey capture is accomplished with the help of a uniquely-formed catching basket. This catching basket is formed by the larval head appendages functioning in concert. Whereas the individual morphological structures have been described in detail, the successive movements and their functional relation, have remained speculative [8].

The role of *Chaoborus* as a ubiquitous predator has been investigated in the context of predator-induced defenses in the small filter-feeding freshwater crustaceans of the genus *Daphnia* Müller (e.g. [9,10,11,12,13,14]). It has been observed that in the coexistence of predators, *Daphnia* exhibits a different morphology than in predator absence, and that these morphologies are predator-specific (for review see [15,16]). For instance, if *Chaoborus* larvae are present, *Daphnia pulex* Leydig develops small cuticular protuberances at the dorsal side of their head, so-called ‘neckteeth’. This has become a textbook example of the function of phenotypic plasticity in predator-prey interactions. While it has been shown that the plastic development of the daphniid’s carapace efficiently enhances its survival [10,17,18], the functional role of these developments is still unclear. This is mainly because the functional morphology of the predator mouthparts and their interaction with the prey have not yet been studied in detail.

To close this gap, we used the predator-prey system of *C*. *obscuripes* larva and *D*. *pulex*. We studied the larval head morphology three-dimensionally using micro-computed tomography and analyzed the kinematics during prey capture with high-speed video recordings of attacks on undefended and defended *D*. *pulex*. We furthermore conducted predation experiments to distinguish between pre- and post-catch effects of *D*. *pulex*’ defenses.

## Material and methods

### Experimental organisms and induction of the daphniids defenses

An age-synchronized culture of *Daphnia pulex*, clone R9 (originating from Canada) was reared under constant conditions in a climate cabinet (day:night cycle of 16:8 hours at 20° C ±0.1° C). The culture was kept in 1 L glass beakers (WECK; Germany) containing charcoal-filtered tap water and was fed with the algae *Acutodesmus obliquus ad libitum*. To ensure stable population growth, animal density was kept below 30 individuals per 1 L. *Chaoborus obscuripes* larvae were captured in ponds of the botanical garden of the Ruhr-University Bochum, Germany, and kept in densities of 50 individuals in 1.5 L beakers at 4°C ±1° C. Larvae were fed daphniids every 48 hours.

For the experiments, female daphniids holding embryos in the fifth stage of development were selected from an age-synchronized culture using a stereo microscope and randomly divided into two groups i.e. ‘undefended group’ and ‘defended group’. The animals were transferred into different 1 L beakers filled with charcoal filtered tap water. In beakers containing the ‘defended group’ a net-cage (mesh width of 100 μm) was inserted. Into that net-cage 10 *C*. *obscuripes* larvae and 100 juvenile *D*. *pulex* R9, as food for the larvae, were added. The ‘undefended group’ was reared likewise in tap water without *C*. *obscuripes* larvae. Both groups were fed with the algae *Acutodesmus spec*. *ad libitum*. After the first molting (approximately 36h after hatching), juveniles of the second instar were removed from the beakers and used in the experiments. Only *C*. *obscuripes* larvae in their 4^th^ stage were used in the experiments.

### High-speed video recordings

We used a Photron Fastcam (SA1.1, Photron, Pfullingen, Germany) with a B18Z06MA-1 F2.5 / 18–108mm 2/3" lens for high-speed video recordings. The catching events were recorded in a miniature aquarium composed of one object glass slide and two glass cover slips glued together with non-toxic dental silicone (Polyvinylsiloxane, Coltène, Switzerland). The object slide served as a base and the two cover slips as aquarium sides, while the two head sides were sealed with dental silicone forming a spacing of 3 mm. One *C*. *obscuripes* larva and three juvenile daphniids were manually added into the miniature aquarium filled with approximately 5 ml of water. In total, 48 catching attempts were recorded (23 using undefended and 25 using defended animals), using the Fastcam viewer software (ver.351, Photron) set on continuous loop recording and post trigger. The first recordings were made with 3000 fps, then after a review, the frame rate was increased to 5000 fps and finally to 8000 fps to avoid motion blur. We analyzed the recordings for interference of the daphniids’ neckteeth with either the *C*. *obscuripes* catching basket or mouthparts, as well as for duration of the catching event and its consecutive steps.

### Micro-CT imaging and 3D reconstruction

To observe the extended catching baskets of *C*. *obscuripes*, individual larvae were fixed between two microscope-slides, and the hemolymphatic pressure was gradually increased by carefully squeezing the microscope-slides together until the free head extended the appendages. In order to retain applied pressure, a thin thread was used to ligate the head. Afterwards the larva was fixed in EtOH 70% and chemically dried. For this purpose, the sample was dehydrated in an ethanol series: 20 min in 80% EtOH, 20 min in 90% EtOH, 20 min in 99% EtOH, 20 min in acetone, and 20 min in acetone/hexamethyldisilazane (HMDS) 50/50 ratio. Finally, the sample was covered with a small amount of HMDS that was permitted to evaporate [19].

The dried sample was mounted on a micro-CT sample holder with a vertically-oriented body axis. The scan was performed with a desktop micro-CT (Skyscan 1172, Bruker micro-CT) with an effective voxel size of 1 μm^3^. Over a 180° rotation, 1440 x-ray images were recorded, which corresponds to steps of 0.125°. Based on these x-ray projection images, an image stack with 1733 16 bit grey scale slice images was reconstructed. The resulting volumetric dataset had a voxel size of 1 μm. This volumetric dataset was visualized and quality checked with CTvox (CTvox 2.7.0, Bruker microCT).

Volume reconstruction of the head capsule, its appendages and the involved muscles was done using the ImageJ distribution ‘FIJI’ [20,21] with the ‘segmentation editor’ plugin (J. Schindelin, F. Kusztos, B. Schmid; Department of Genetics and Neurobiology Würzburg). Using the ImageJ selection tools, the respective tissues were marked within successive slices of the image stack and refined with the threshold tool based on grey values. This process was repeated for every third to sixth slice, depending on the degree of structural variability. The slices in between were automatically labelled via interpolation. Afterwards, a surface mesh based on the reconstruction was exported as an *.stl file and used for the animation of the catching event.

### Surface mesh and animation

The animation was created with the open source 3D computer graphics software Blender (Blender 2.72b, Blender foundation, Amsterdam, the Netherlands) following the instructions of Garwood and Dunlop [22].

The surface mesh was loaded into the Blender software. To optimize computational effort, we reduced the number of vertices using the “decimate modifier” function. Subsequently, the surface was smoothened in “sculpt mode” using the “sculpt draw brush”, while permanently comparing the surface mesh to the original micro-CT-data to minimize computational bias. We only used the external surfaces from the volume reconstruction to maintain natural appearance.

For animation of the head appendages, so-called “bones” were used in the Blender software. These “bones” allowed the animation of individual appendages involved in the catching event. Every “bone” was associated with the respective appendage of the surface mesh and several “bones” were combined to form one movable element. To define the vertices of the surface mesh associated with a specific bone, the “weight paint mode” was used. The animation of the catching event was modelled by defining key positions that occurred during the movement of the appendages. To smooth the animation, the movement between key positions was interpolated and rendered into a video.

### Predation trials

In order to be able to differentiate between pre- and post-attack defenses in *D*. *pulex*, we conducted predation trials as described by Havel and Dodson [10]. In contrast to the high-speed video recordings we recorded a predefined time span (60 min) aiming for multiple attack events per replicate. We illuminated a glass tank (12cm x 1.5cm x 10cm) with diffuse light. Black cardboard was positioned behind the aquarium for contrast enhancement of transparent *D*. *pulex*. Experiments were conducted with one starved *C*. *obscuripes* larva and 20 *D*. *pulex* juveniles in the second instar (either undefended or defended) This setup was monitored for one hour. Larvae that either did not strike within the first 10 minutes, or made fewer than two successful catches during the 60 minutes, were exchanged by another unfed larva. Every consumed *D*. *pulex* was replaced by a new one to keep prey density at a constant level. An SLR camera (Nikon D5100, Nikon Corporation, Tokyo, Japan) with a 60 mm / f 2.8 lens in automatic mode was used for video recording (30 fps). To analyze the videos, strikes, evasions, contacts, escapes and ingestions were all counted (Table 1). These conditions were defined according to Havel and Dodson [10]: a strike was defined whenever *C*. *obscuripes* grasped towards a daphniid; every strike resulted in either contact or evasion. Whenever a *C*. *obscuripes* head touched a daphniid, a contact was noted. Every strike without contact was classified as evasion. Contacts resulted in either escape or ingestion. The experiment was replicated 11 times per treatment group (defended daphniids/undefended daphniids). Data was analyzed with R and visualized with the library ggplot2 [23,24]. Data was found to follow a normal distribution using a Shapiro-Wilk test and was thus analyzed using a Student’s t-test between defended and undefended *D*. *pulex*. In accordance, we compared the number of strikes as well as the contacts per strike and the ingestions per contact.

**Table 1:** Definition of terms used for the analysis of predation success.

Term	Definition
Strike	Attack movement towards the prey item.
Evasion	Attack movement does not lead to a contact between any head appendage (catching basket) and prey item.
Contact	Attack movement leads to a contact between any head appendage (catching basket) and the prey item.
Escape	Contact does not lead to prey consumption i.e. prey item escapes after contact
Ingestion	Contact leads to consumption

## Results

### Head morphology

**Fig 1:**
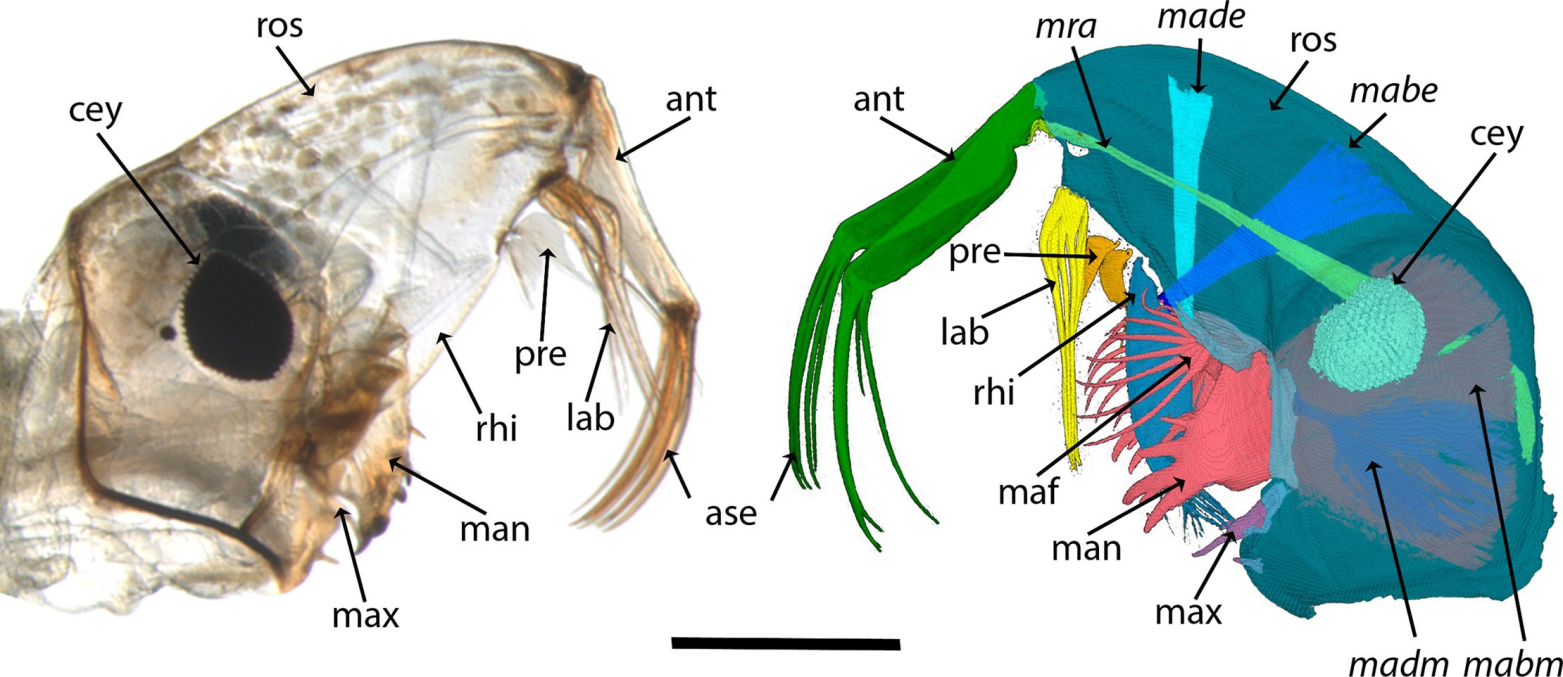
Overview of *Chaoborus obscuripes* larval head morphology. left: microscopic image, right: micro-CT based reconstruction. ant = antenna basis; ase = antenna’s setae; cey = compound eye; lab = labral setae; mabe = *musculus abductor epipharyngis*; mabm = *musculus abductor mandibulae*; made = *musculus adductor epipharyngis*; madm = *musculus adductor mandibulae*; maf = mandibular fan; man = mandible; max = maxilla; *mra* = *musculus retractor antennae*; pre = prelabral appendages; rhi = rhinopharynx; ros = rostrum. Scale bar = 500 **μ**m.

*Chaoborus* larvae are eucephal and therefore possess a sclerotized head capsule with differentiated head appendages, including antennae and a complete set of mouthparts i.e. labrum, mandibles and maxillae (Fig 1). Some of these structures, e.g. the rhinopharynx and labrum, are strongly modified in comparison to a general insect form. The larval head capsule of *C*. *obscuripes* anteriorly elongates into a laterally-flattened rostrum. Paired antennae originate from the tip of the rostrum. The labrum originates on the ventral side of the rostrum posterior from the antennal joint. The labrum is highly modified into pairs of bristles. Posterior from the labrum (labral setae), there are the pre-labral appendages, two leaf- or blade-shaped structures that reach ventrally into the space in front of the mouth opening. Further posteriorly, in front of the mouth opening, connects the massive rhinopharynx. The head appendages directly associated with the mouth are the mandibles, each wearing a fan of bristles, and the rather minute maxillae.

We created a high-resolution 3D image of the *C*. *obscuripes* larval head morphology from a micro-CT scan. By using the scan, we were able to reconstruct the volume of the head capsule including all appendages and their associated muscles. We also reconstructed one compound eye as representative for the visual organs (Fig 1). Here, we describe the head morphology in detail based on this reconstruction.

### The antennae

The antennae of the *C*. *obscuripes* larva are modified from the insect body plan into grasping organs. They originate from the tip of the elongated rostrum. These modified antennae have a first segment that is nearly as long as the rostrum, and each possesses four proximally-curved setae (Fig 1). Our micro-CT data show that one pair of muscles is associated with the antennae: the *musculi retractor antennae*. They arise as a strong fiber bundle from the head neckline and insert ventrally at the respective antenna base (Fig 1). In resting position, the antennae are flexed in approximately 90° to the ventral side of the rostrum. The antennae setae are positioned rostral pointing ventrally and covering the mouth opening (Fig 1).

### The labral setae and prelabral appendages

The labrum is modified from the insect body plan into five pairs of bristles and thus called labral setae. They originate at the ventral side of the rostrum between the mouth opening and the antennae (Fig 1).

The prelabral appendages originate posteriorly from the labral setae. They are partially covered by the labral setae, if the latter are in resting position (Fig 1). Their appearance is blade-like, which led to their descriptive name “knife hairs” (Messerhaare) [8].

### The mandibles

**Fig 2:**
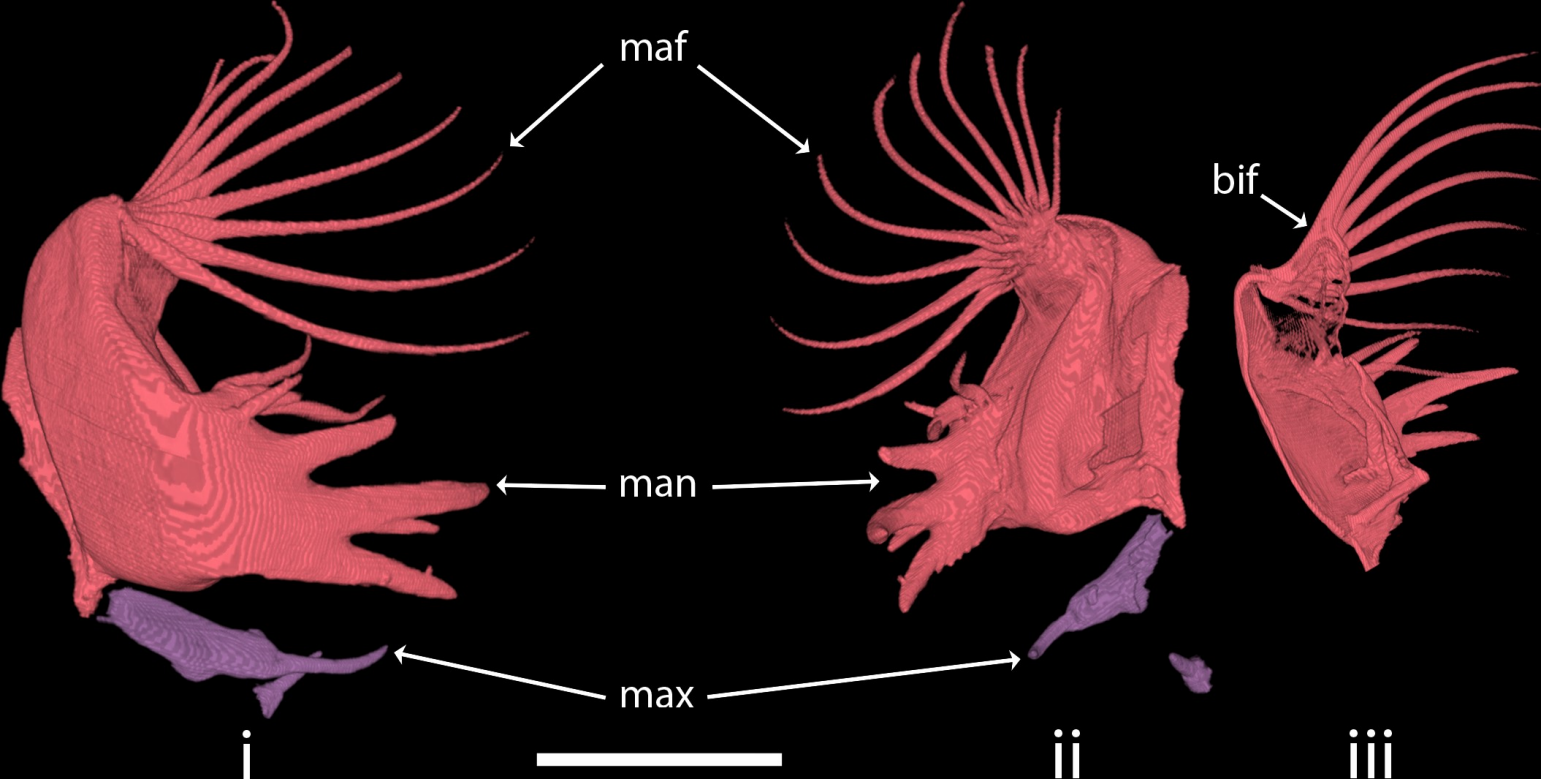
Reconstruction of *Chaoborus obscuripes* larva’s mandibles and maxillae in ventral (i) and proximal (ii) views and in cross section (iii). bif = bifurcation of the fan bristle bases; man = mandible; maf = mandibular fan; max = maxilla. Scale bar = 200 **μ**m.

The robust mandibles end as sharp sclerotized spikes at the ventral anterior tip (Fig 2). These six spines differ in shape and therefore can be subdivided into two groups. Two thin dorsally-laying spikes have a bristle-like appearance and insert into a membranous proximal surface of the mandibles. The other four spikes are robust and immobile. The longest of these spikes bifurcates into an additional smaller tip.

Two pairs of muscles are associated with the mandibles. The *m*. *adductor mandibulae* is massive and consists of three strands, two originate dorsally from the compound eye and one originates from the postoccipital rail (Fig 1). All three strands insert inferiorly to the middle of the proximal mandible edge. The *m*. *abductor mandibulae* originates dorsally from the compound eye and inserts at the middle of the distal edge of the mandible.

### The mandibular fan

The mandibular fan originates at the mandibular dorso-posterior edge and it is characterized by ten bristles (Fig 2). Each bristle is bifurcated at its base. The dorsal branch of the bristle inserts at the membranous proximal part of the mandible, whereas the ventral branch articulates on the robust ventral edge of the mandible.

### The rhinopharynx

**Fig 3:**
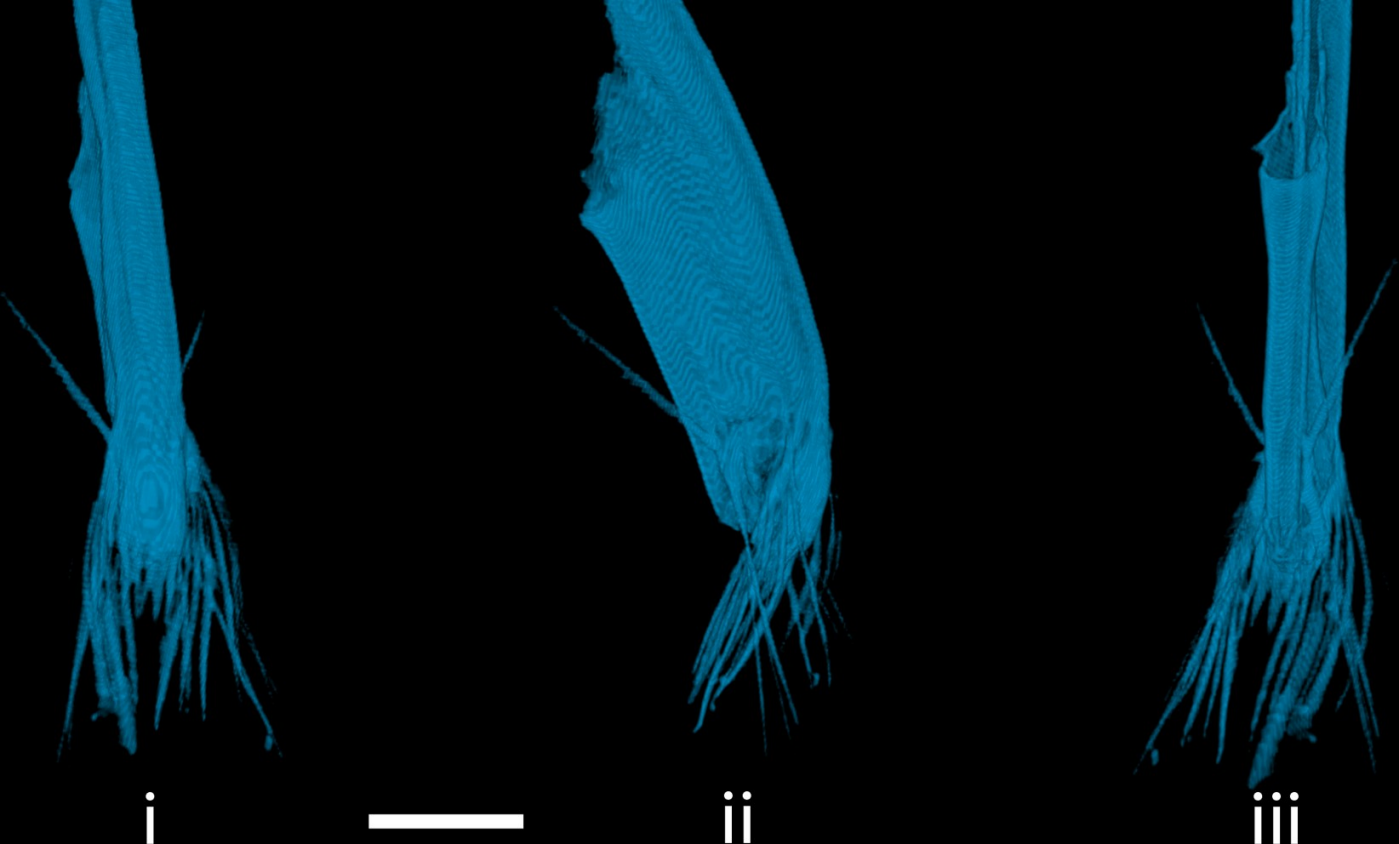
Reconstruction of the rhinopharynx of *Chaoborus obscuripes* larva in anterior (i) lateral (ii) and posterior (iii) view. Scale bar = 100 **μ**m.

The finger-shaped rhinopharynx protrudes at an angle of about 35° from the rostrum in between the prelabral appendages and the mouth (Fig 1). It has a laterally-flattened appearance (Fig 3). Two muscles are associated with the rhinopharynx: *m*. *abductor epipharyngis* and *m*. *adductor epipharyngis* (Fig 1). The rhinopharynx’ tip is laterally studded with symmetrically arranged immobile bristles: four bristles arise on each side at the posterior part of the rhinopharynx tip; whereof one points to posterior/superior direction, and the other three bristles point inferiorly. Apical on each side, there is an almost vertical row of five bristles which are opened in a fan-like manner and point anteriorly/inferiorly. The rhinopharynx tip ends anteriorly in a row of six spiky squamous fork-like thorns (Gabeldornen) that are accompanied by five smaller bristle scales (Borstenschuppen) with an offset in the basally direction. Posteriorly, the tip has a concave shape.

### Strike kinematics

**Fig 4:**
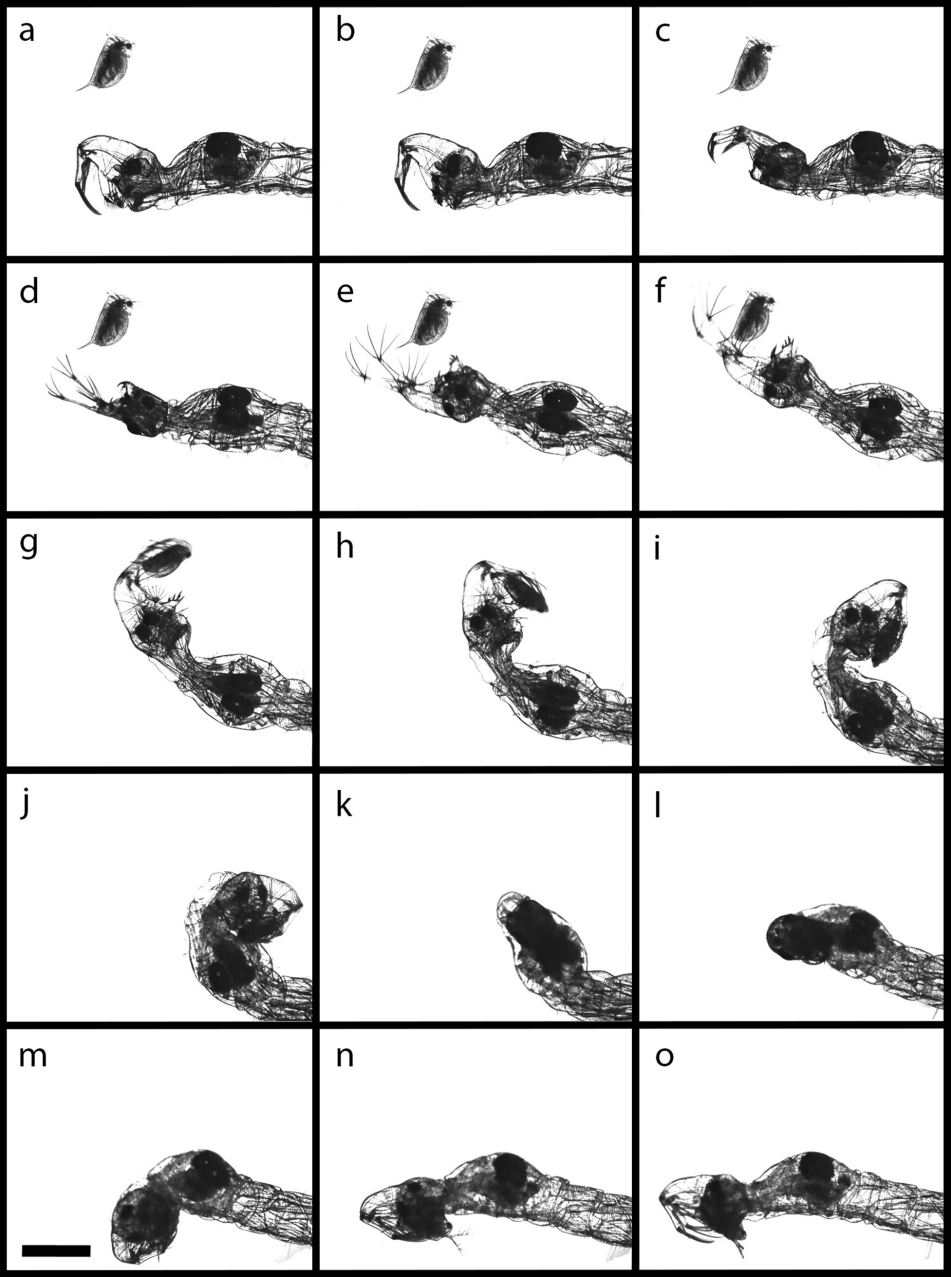
Time series of a *Chaoborus obscuripes* catching event. Larva preys on a defended *D*. *pulex* (8000 fps). a) -29.1 ms b) 0 ms c) 3.9 ms d) 6,2ms e) 8 ms f) 9.7 ms g) 12.7 ms h) 14.9 ms i) 21.8 ms j) 27.9 ms k) 50.9 ms l) 83.4 ms m) 117 ms n) 181.4 ms o) 280.8 ms. Scale bar = 1000 **μ**m. See supplementary video (S1 Movie).

As an ambush predator, *Chaoborus* rests in the water column waiting for prey to enter its striking distance [25]. Therefore, each catching event starts from a resting position of the larva. In this resting position, the antennae are relaxed, pointing ventrally while their setae are positioned in a parallel orientation, the labral setae also point ventrally and recline in parallel orientation, the rhinopharynx is positioned in front of the mouth opening, the mandibles are closed and their fans are folded towards the mandibles’ proximal side (Fig 4A; t = -29.1 ms). The catching process starts immediately after the *C*. *obscuripes* larva detects a daphniid. The first step of the motion sequence is a retraction of the rhinopharynx towards the head (Fig 4B, t = 0 ms). This is followed by a set of nearly simultaneous, uniform movements i.e. the mandibles, the antennae, including their setae, (t = 9.99 ms ± 1.64 s.d., n = 10) and the labral setae (t = 10.46 ms ± 1.69 s.d., n = 10) open and form the catching basket. The catching basket is then oriented in the direction of the prey by rotation of the whole larva along its body axis and a bending movement of the head towards the thorax (Fig 4C–4E). Then, just before prey contact, the mandible fan opens and obstruct prey escape in posterior direction (Fig 4G; t = 12.4 ms ± 1.7 s.d., n = 10). In most cases, the prey contact (t = 13.87 ms ± 2.1 s.d., n = 10) is conducted by antennae. The antennae and their setae retract (t = 15.41 ms ± 2.43 s.d., n = 10) which moves the prey in the direction of the mouth (Fig 4H). The larval head continues its radial movement towards the prey until the catching basket presses the captured prey against the larval thorax (Fig 4I and 4J; t = 27.42 ms ± 7.43 s.d., n = 10). Finally, the larva returns into its resting position (t = 267.9 ms ± 296.21 s.d., n = 10) and the prey handling is characterized by an alternating stuffing motion of the mandibles. The whole catching process has a duration of less than 300 ms. The folding of the head towards the body and back into resting position takes the longest time (about 270 ms), while the duration from starting the catch to grasping the prey (i.e. the actual strike) only takes around 30 ms (also see S1 Movie).

### Head appendages involved in the catching basket

**Fig 5:**
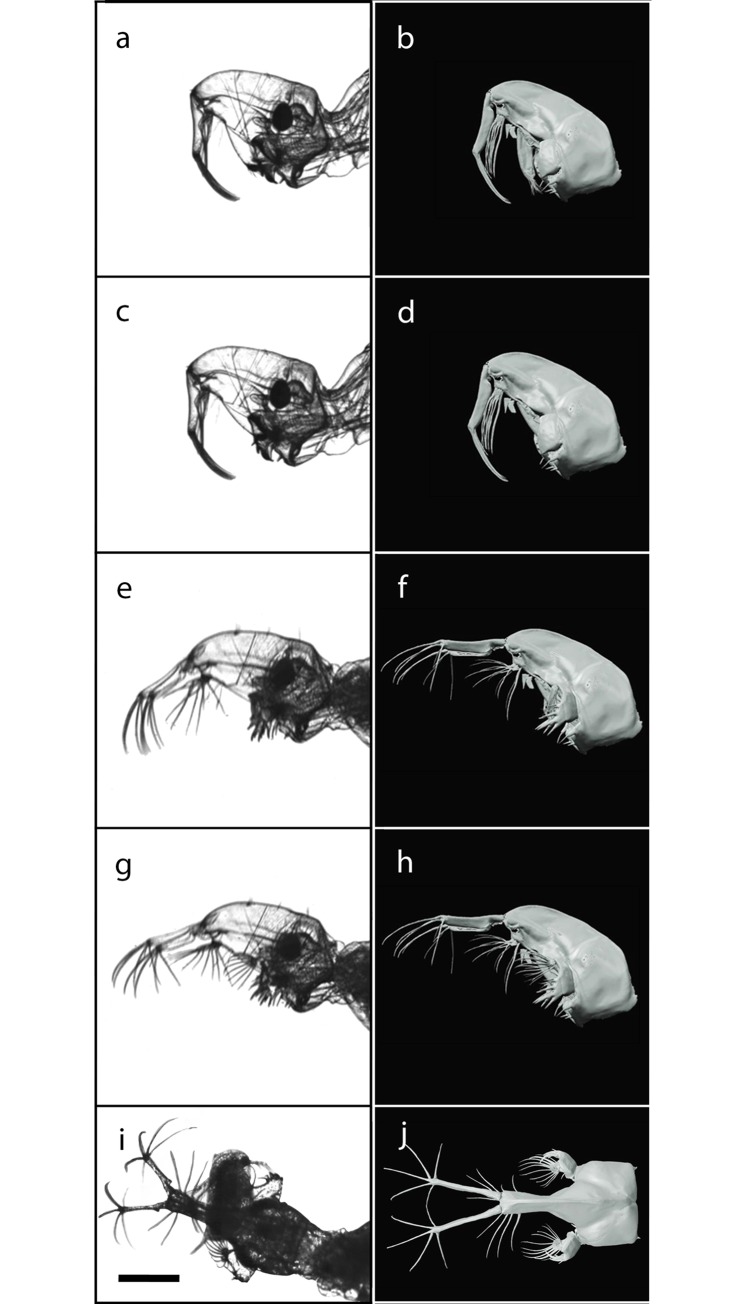
The animated surface model (b, d, f, h, j) of the *Chaoborus obscuripes* larva shown with the corresponding frames from high-speed recordings (a, c, e, g, i), displayed *D*. *pulex* with defensive neckteeth (i). Scale bar = 500 **μ**m.

During the attack movement, the involved appendages form an effective catching basket (Fig 5). The anterior part of this basket is realized by the antennae and their widely-spread setae. The posterior side is formed by the mandibles and the mandibular fans. The space between these two elements is closed by the laterally spread labral setae. The detailed movement and mechanisms of every part of the catching basket are explained in the following sections.

#### Antennae

The lifting movement of the antennae is most likely a result of increasing hemolymph pressure, as the antennae only possess retractor muscles. The antennae insert at the rostrum through a flexible joint. A membranous joint skin is situated ventral to this joint: it expands when hemolymph pressure increases and lifts the antennal basis. The articular cavity of the antennal joints encloses the articular head more lateral than superior and inferior. The rotary axis is not orthogonal to the sagittal plane, but slightly leaned dorsally. Accordingly, the extended antennae are forked laterally at an angle of 25° (Fig 5I and 5J). The retraction movement executed after prey contact is performed with the *musculus retractor antennae*.

#### Labral setae

It can be seen in the high-speed videos that the labral setae are spread during attack movement (Fig 5E and 5F). The labral setae can be divided into two groups: the anterior four pairs and a posterior pair. When spread, the two groups point in different directions. In this moment, the gap between antennae and mandibles is almost completely closed.

#### Mandibles

When mandibles are retracted into resting position, the bristles of the mandibular fan rest in parallel alignment (Fig 5E and 5F). When the mandibles are opened, they erect splayed to a fan (Fig 5G and 5H).

After successful prey handling, the mandibles stuff the unchewed prey into the esophagus by alternating movements. Although these movements appear to have no shredding function, sometimes daphniids burst during mandibular manipulation.

### Animation of the catching event

**Fig 6:**
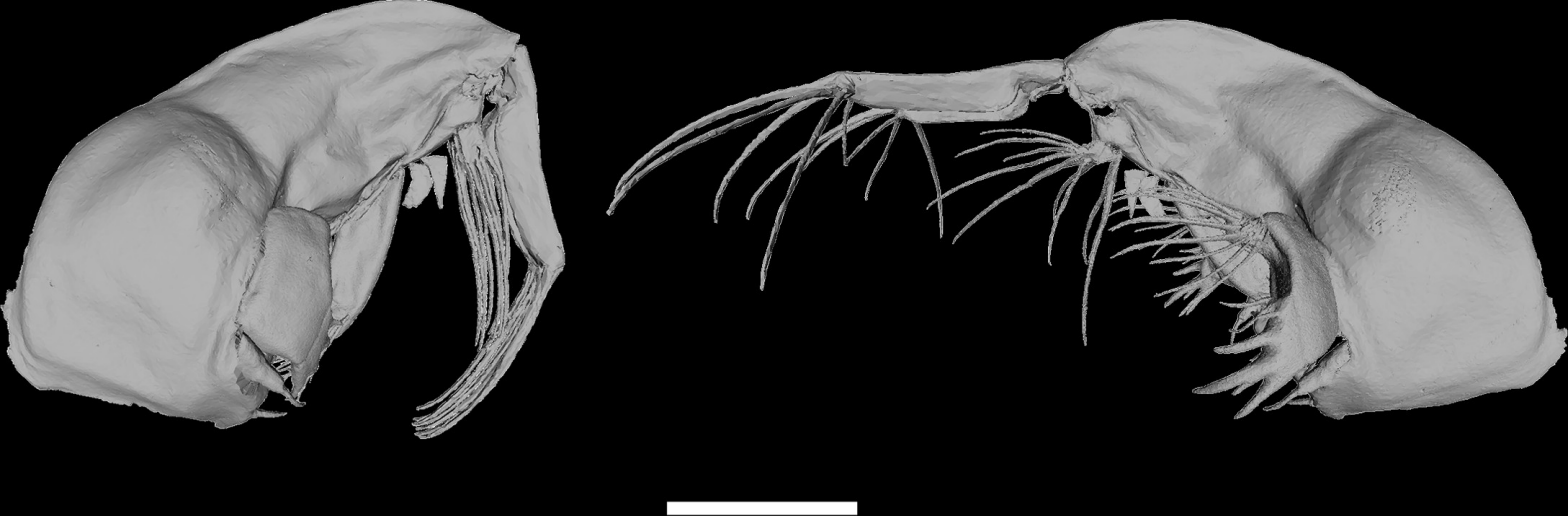
**Surface model of the *Chaoborus obscuripes* larval head capsule** with closed (left) and opened (right) catching basket. Scale bar = 500 **μ**m.

Based on our reconstruction of the head capsule, we created a surface model for the outer morphology i.e. the head capsule with all appendages (Fig 6, S1 and S2 Figs). With the conducted high-speed video recordings, the motion sequence of the involved head appendages was identified, including their maximal positions in both resting and attacking positions. This data are synchronized to the surface model and resulted in a complete animation of the catching event (Fig 5B, 5D, 5F, 5H and 5J, S2 and S3 Movies).

### Efficiency of the catching basket

In our analysis of the high-speed video recordings, we found a tendency of less successful attacks by *C*. *obscuripes* on defended (success rate of ~50%) than on undefended (~80%) prey, however, no significant differences were found (Chi-square test, χ^2^ = 3.0662, n_undefended_ = 23, n_defended_ = 25, p = 0.08).

**Fig 7:**
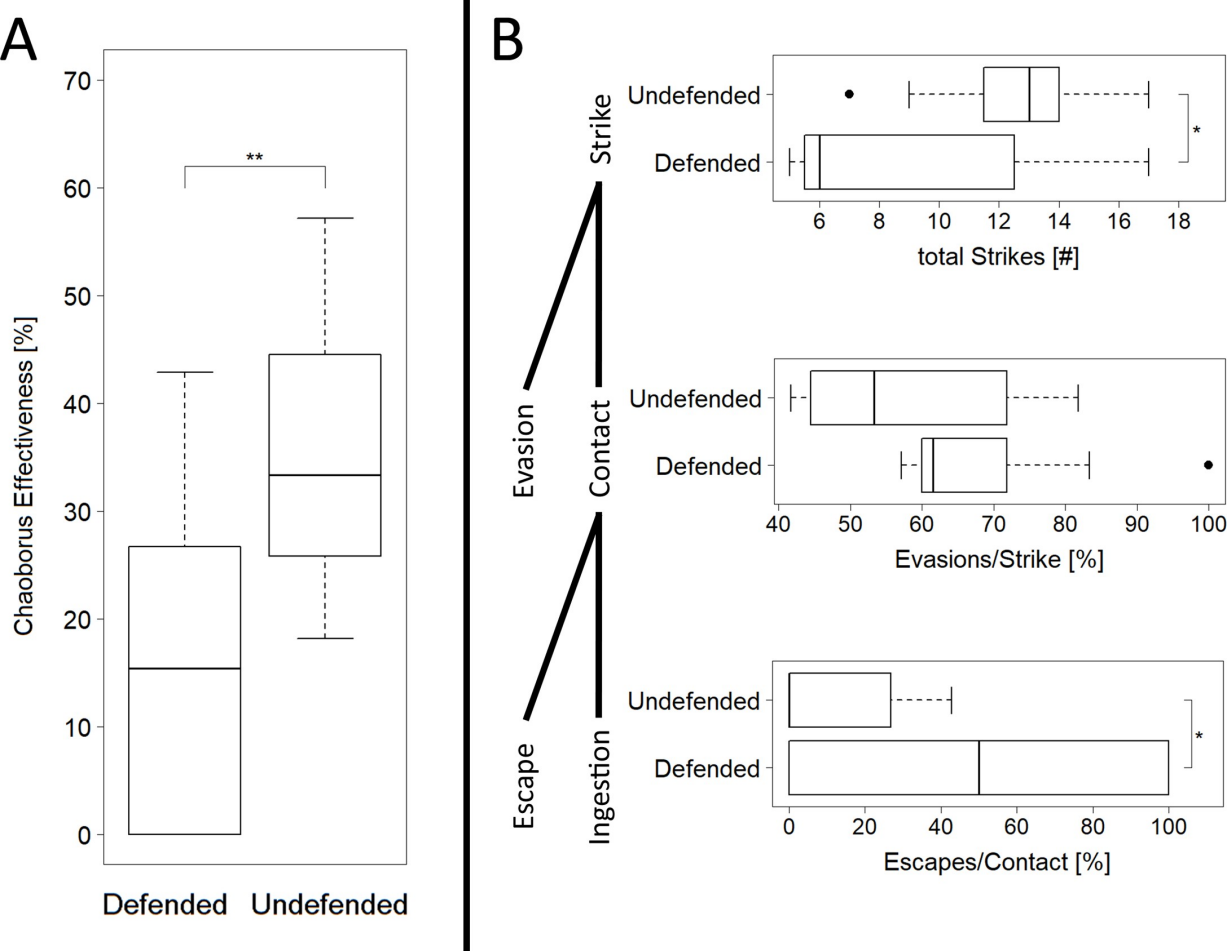
***Chaoborus obscuripes* catching efficiency** (n = 11, t-test, level of significances: p<0.05 ‘*’, p<0.01 ‘**’, p<0.001 ‘***’) A) *O*verall prey capture efficiency of larvae in the predation experiments B) Detailed analysis of predatory efficiency at different steps during the predation event. All boxplots show median, interquartile range (IQR) as box and 1.5xIQR as whiskers.

The feeding experiments revealed a significantly lower success rate of *C*. *obscuripes* if facing defended prey (t-test, t = 3.12, n = 11, p = 0.006; Fig 7A). The number of strikes by *C*. *obscuripes* on defended prey also was significantly lower than on undefended prey (t-test, t = 2.5462, n = 11, p = 0.02; Fig 7B). During the strike, no significant difference was found for the proportion of evasion events (t-test, t = 1.7034, n = 11, p = 0.1; Fig 7B). After contact, during prey handling, a significant difference between undefended and defended prey was observed. Defended daphniids escaped more often than undefended (t-test, t = 2.6761, n = 11, p = 0.02; Fig 7B).

## Discussion

### *Chaoborus* catching movement

Here, we provide a detailed 3D animation of the *Chaoborus* larval head performing the motion sequence during daphniid capture. This animation was created using high-speed video recordings and micro-CT data. Our results give insights into the catching event with a high time and spatial resolution and confirm many of the hypothesized movements and functions of the larval catching basket components previously made by Schremmer [8]. However, the speculation that the labral setae demonstrate insignificant movability due to the absence of muscles was not confirmed. In fact, they spread open widely, presumably due to changes in the hemolymph pressure. The spread labral setae form the lateral sides of the catching basket between antennal setae (anterior side of the catching basket) and the mandible fan (posterior side). The latter appears to open due to changes in hemolymph pressure as well. We did not find any attached muscles but a membranous field on which a branch of each of the fan’s bristles rests. A convex distortion of this membranous field would thereby lever the bristles into an upright position. Schremmer [8] anticipated that the rhinopharynx is involved in prey ingestion and furthermore prevent prey escape during handling. No movements that support this hypothesis were observed in any of our recordings. In fact, the rhinopharynx appears to initiate the attack by a folding movement towards the mouth opening. It stays in this neutral position during prey capture, handling, and even ingestion. Thus, we do not anticipate its involvement in neither prey handling, ingestion nor escape prevention. Since the retraction of the rhinopharynx is always the first movement of an attack, it may be involved in the spreading of the antennae and the several setae participating in the formation of the catching basket. We assume that the retraction of the rhinopharynx elevates the hemolymph pressure in the head capsule by reducing its internal volume, and thereby this forces the antennae and setae to spread. The two prelabral appendages are misleadingly designated as “Messerhaare” (knife hairs), since they have a blade like shape. They are used as important characteristics for species identification. However, they are not involved in prey handling or ingestion. They may have sensory functions, but so far this hypothesis is not confirmed.

With an overall duration of 14 ms from start of the movement to prey contact, the attack of *Chaoborus* larvae ranks within the fastest known attack movements in the animal kingdom. The praying mantis *Coptopteryx viridis* (Mantodea, Mantidae) attacks within 42 ms [26], and for mantis shrimps *Squilla empusa* (Stomatopoda, Squillidae) and *Hemisquilla ensigera* (Stomatopoda, Hemisquillidae) attack movements within 4–8 ms have been reported [27]. The fastest strikes have been reported for trap-jaw ants of the genus *Odontomachus* (Hymenoptera, Formicidae) and some species of trap-jaw spiders (Araneae, Mecysmaucheniidae), which can both accomplish a full strike in less than 1 ms [28,29].

High strike velocity and performance can be explained by arms race dynamics, where there is a reciprocal enhancement of selective pressure on predatory and escape performance [30]. For instance, in soil communities, rove beetles (Coleoptera, Staphylinidae) and some harvestmen evolved ultrafast-strikes of sticky appendages to capture springtails (Collembola), which can avoid attacks by propelling themselves into the air within a few milliseconds by using a power-amplified spring organ [31,32]. Likewise, there are predatory devices that capture zooplankters with ultrafast movements, like the specialized leaves of the bladderwort plant, which hold the record of the fastest movements among plants [33]. Considering the maximal reported swimming speed of *D*. *pulex* is 15 mm/sec [34] a daphniid can cover a distance of 300 μm within the timeframe of a larva’s attack. This is less than half the body length of the juvenile prey. In case of the *Chaoborus* larvae, even if attack and escape start simultaneously, a successful active escape through swimming is unlikely and was not observed in any of the high-speed recordings, which underlines the efficiency of the *Chaoborus* larval catching basket. Considering the difference in prey swimming velocity and predator attack speed the latter does not appear to be optimized for this particular prey. Most likely, this attack speed is an adaptation for catching copepods that have swimming velocities of 102 mm/sec average maximum speed and co-occur in the same habitats [35].

### Defenses by *D*. *pulex* against attacks of *C*. *obscuripes* larvae

The analysis of high-speed recordings did not allow us to elucidate how neckteeth interfere with *Chaoborus’* predation sequence. In none of the recorded catching events an interference or interaction of the neckteeth with the predators’ capture appendages or mouthpart was identifiable. At the same time, our feeding experiments, still confirm, that neckteethed *D*. *pulex* are better defended (also reported by [10,13,17,18]). Furthermore, the predator efficiency observed in the high-speed video recordings affirms the hypothesis of the mechanical interference of the neckteeth with *Chaoborus’* mouthparts or catching basket components. The capture efficiency in the high-speed video recordings showed a non-significant trend to be lower for the defended prey (Chi-square test, p = 0.08) and, because of the experimental setup, only reflects post attack defensiveness. Thus, the above mentioned interference-hypothesis is supported, although such an interference was not visually observable in any of these recordings [17,36]. Since the feeding experiments revealed defensive effects before as well as after predator-prey contact, *D*. *pulex* seems to possess a complex set of defenses including predator avoidance and post- contact defenses. Similarly, helmets in *D*. *cucullata* have been shown to enhance evasion and escape probabilities against *Chaoborus* larvae predation [37]. Those helmets are discussed to reduce the water turbulences while swimming and thereby confuse haptic sensing of *Chaoborus* larvae potentially resulting in a misjudgment of prey distance [37]. For the rather inconspicuous neckteeth of *D*. *pulex* however, such a hydrodynamic effect seems unlikely. Therefore, behavioral defenses realized in reduced activity and thus reduced contact probability may be the main factor of increased pre-contact defensiveness [38]. This hypothesis has been supported by Weber and van Noordwijk [39]. The main post-contact defense remains unknown, since we did not observe any interference of the neckteeth with the larval catching basket in the high-speed video recordings. An additional factor that could contribute to an increased defensive effect possibility is the larval size in our experiments, since not only 4^th^ instar, but also 3^rd^ instar larvae prey on daphniids. Therefore, neckteeth may additionally protect against 3^rd^ instar larvae. In our high-speed video recordings, we could not observe the mechanism by which neckteeth mechanically interact with mouthparts of *Chaoborus* larvae, which might be due to the fact that prey item and larvae’s head appendages visually overlap in the critical moment.

## Conclusion

With the presented 3D animation, we illuminate details of the *Chaoborus* larval capture movement, when hunting for *D*. *pulex*, a crucial step in this well-studied predator-prey relationship. We demonstrated that the use of state-of-the-art 3D-imaging methods in combination with high-speed kinematic analyses are suitable to explore the biomechanics of predator-prey interactions. We hope that with our contribution to the *Chaoborus*-*Daphnia* predator-prey relationship, we spark some ideas for further studies and add a step to the unraveling of *D*. *pulex* ‘neckteeth’ mode of function.

## Supporting information

S1 Fig Surface mesh (.stl) of the *Chaoborus* larva head morphology with closed catching basket. (STL)

S2 Fig Surface mesh (.stl) of the *Chaoborus* larva head morphology with opened catching basket. (STL)

S1 Movie High-speed footage of a *Chaoborus* larva attacking a defended daphniid. (MP4)

S2 Movie Animation of the opening catching basket of *Chaoborus* larvae based on micro-CT data and high-speed footage. (AVI)

S3 Movie Animation of the opening catching basket of *Chaoborus* larvae based on micro-CT data and high-speed footage, multiple angles of view. (MOV)
